# Spontaneous closure of patent ductus arteriosus after an episode of Kawasaki disease: a case report

**DOI:** 10.1186/1752-1947-6-36

**Published:** 2012-01-25

**Authors:** Ming-Chih Lin, Yun-Ching Fu, Sheng-Ling Jan

**Affiliations:** 1 Department of Pediatrics and Institute of Clinical Medicine, National Yang-Ming University, Taipei, Taiwan; 2 Division of Pediatric Cardiology, Department of Pediatrics, Taichung Veterans General Hospital, Taichung, Taiwan; 3 Institute of Epidemiology and Preventive Medicine, National Taiwan University, Taipei, Taiwan

## Abstract

**Introduction:**

Kawasaki disease is regarded as systemic vasculitis. Many experts believe that not only coronary arteries but also other small arteries are involved during the period of systemic inflammation. However, the evidence to support this point view is limited.

**Case presentation:**

We report the case of a one-year-four-month-old Taiwanese girl whose patent ductus arteriosus was incidentally found during an episode of Kawasaki disease. The ductus closed spontaneously after the acute phase of Kawasaki disease.

**Conclusions:**

In this patient, the patent ductus arteriosus may have closed spontaneously after Kawasaki disease due to its involvement in the generalized vasculitis that this disease incurs. This would support the theory that the vasculitis of Kawasaki disease is limited not only to coronary arteries but also to all medium- sized arteries.

## Introduction

Kawasaki disease is regarded as systemic vasculitis [1]. Many experts believe not only coronary arteries but also other small arteries are involved during the period of systemic inflammation [1-4]. However, the evidence to support this point view is limited [1,5,6]. We report the case of a one-year-four-month-old girl whose patent ductus arteriosus closed spontaneously after an episode of Kawasaki disease.

## Case presentation

**Figure 1:**
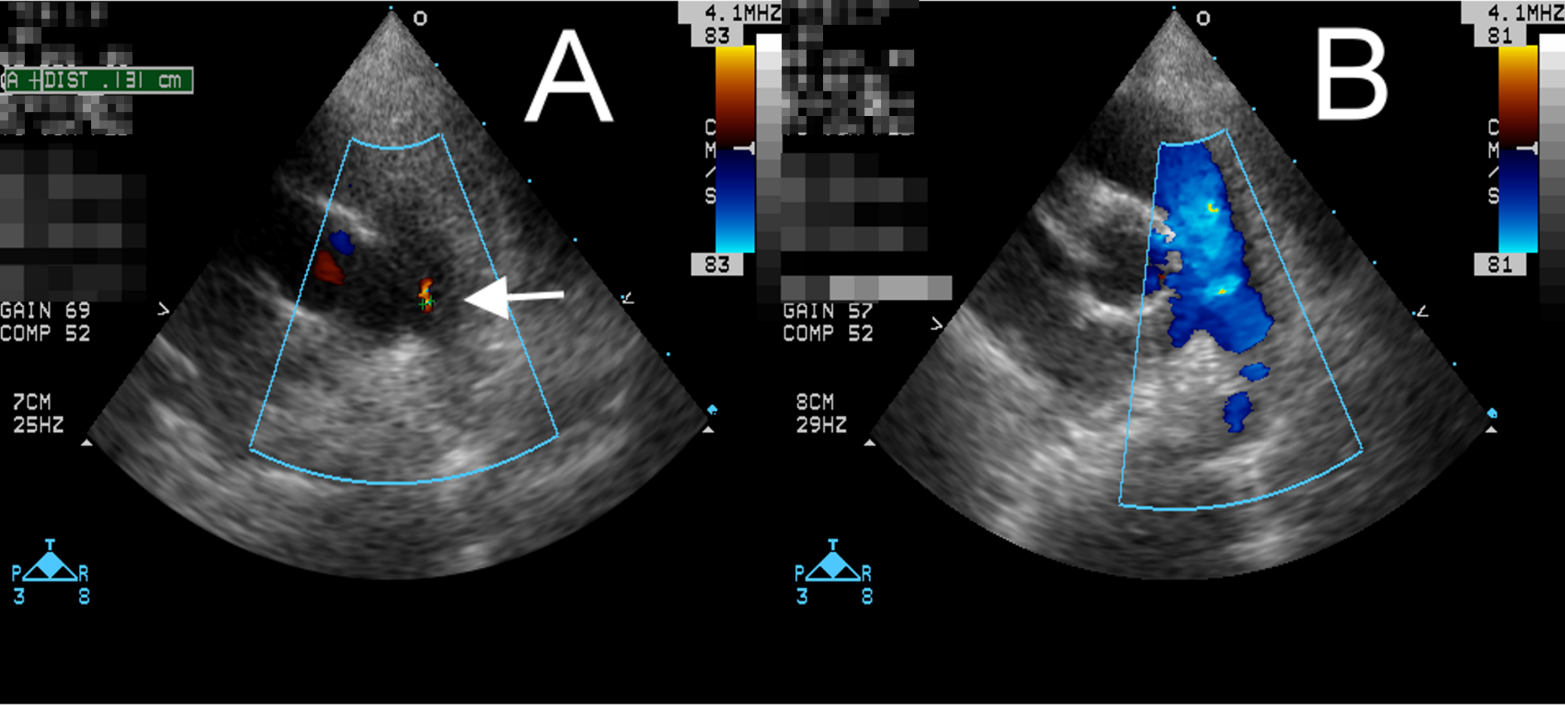
**(A) A tiny patent ductus arteriosus incidentally found during the acute phase of Kawasaki disease (white arrow)**. **(B**) no patent ductus arteriosus found one month after two courses of intravenous immunoglobulin.

A one-year-four-month-old Taiwanese girl was transferred to our hospital from a regional hospital due to prolonged spiking fever for more than three days. Initially, she presented with fissured lips, skin rashes over her trunk and thighs, as well as erythematous and indurative feet. However, neither a neck lymph node greater than 1.5 cm nor conjunctivitis could be found. No heart murmur was heard during auscultation. Laboratory evaluation revealed elevated C-reactive protein at 4.5 mg/dL, a white cell count of 8500 × 10^9^/L, platelet count of 258,000 × 10^9^/L, hemoglobin 12.7 g/dL, albumin 3.8 mg/dL, alanine aminotransferase 16 U/L, aspartate aminotransferase 41 U/L, and mild pyuria (5 to 10 white cells under high power field). Incomplete Kawasaki disease was diagnosed based on echocardiographic findings including perivascular brightness, lack of tapering of the coronary arteries, mild mitral regurgitation, and mild pericardial effusion. When performing echocardiography, a tiny patent ductus arteriosus was incidentally found (Figure 1A). On the sixth day of fever, the first course of intravenous immunoglobulin (IVIG) 2 gm/kg was delivered. However, fever flared up on the seventh day of illness. Thus, a second dose of IVIG was given. The fever subsided thereafter. No coronary aneurysm was found throughout the hospital course. Peeling of fingers was observed in the second week of this acute episode. We did not prescribe indomethacin or ibuprofen for her. One month after the acute episode, follow up echocardiography showed that the patent ductus arteriosus had closed spontaneously (Figure 1B). Closure of the patent ductus arteriosus was confirmed by sequential echocardiography at two, five and 12 months after the Kawasaki disease episode.

## Discussion

Kawasaki disease was first reported in Japan in 1967 [1,7]. It has been reported among children of all races [1]. It is an acute and self-limited vasculitis occurring predominantly in infants and young children. The clinical presentation includes fever, bilateral non-exudative conjunctivitis, erythema of lips and oral mucosa, changes in the extremities, and cervical lymphadenopathy [1]. If left untreated, coronary artery aneurysms or ectasia which may lead to coronary stenosis or total occlusion, myocardial infarction, ischemic heart disease, or sudden death, can happen in 15% to 25% of patients [1,7,8]. The etiology is still unknown. In most industrialized countries, Kawasaki disease is now the most common acquired heart disease among children. No specific laboratory test can assist physicians in establishing the diagnosis. Intravenous immunoglobulin and aspirin can effectively treat this disease [1,7].

Because of a lack of tissue specimens, the literature reporting pathological changes in Kawasaki disease is limited [2-5,9,10]. Most of those reports focused on the inflammation of the coronary arteries. The inflammation could also trigger subsequent remodeling processes in the coronary artery lesions and could continue for years [2,9,10]. This phenomenon could also partially explain the pathogenesis of coronary artery lesions of Kawasaki disease. In the meantime, the inflammation of Kawasaki disease is not limited only to coronary arteries but also affects other medium and small size arteries [2-5]. The evidence supporting this point of view is very limited. Although aneurysms of celiac, mesenteric, femoral, iliac, renal, axillary and brachial arteries have been reported, whether generalized arteritis exists in every patient is still a controversial issue [1,4,6,11]. In our patient, the patent ductus arteriosus may have closed spontaneously after Kawasaki disease due to its involvement in the generalized vasculitis that this disease incurs. This would support the theory that the vasculitis of Kawasaki disease is not only limited to coronary arteries but also to all medium- sized arteries. Spontaneous closure of patent ductus arteriosus might be caused by the prolonged inflammation that necessitated the second course of IVIG. The timing of spontaneous closure is compatible with the disease process, in which active inflammation persisted over several weeks to months followed by progressive fibrosis with scar formation [1,2].

## Conclusion

To the best of our knowledge, spontaneous closure of patent ductus arteriosus in patients with Kawasaki disease has never been reported before. In our patient, the patent ductus arteriosus may have closed spontaneously after Kawasaki disease due to its involvement in the generalized vasculitis that this disease incurs. This would support the theory that the vasculitis of Kawasaki disease is limited not only to the coronary arteries but also to all medium- sized arteries.

## Abbreviations

IVIG: intravenous immunoglobulin.

## Consent

Written informed consent was obtained from the patient's next-of-kin for publication of this case report and any accompanying images. A copy of the written consent is available for review by the Editor-in-Chief of this journal.

## Competing interests

The authors declare that they have no competing interests.

## Authors' contributions

MCL wrote the manuscript. YCF reviewed the article. SLJ provided his experience in caring for patients with Kawasaki disease in the discussion section. All authors read and approved the final manuscript.
